# Undergraduate radiology teaching from the student’s perspective

**DOI:** 10.1007/s13244-012-0206-8

**Published:** 2012-12-07

**Authors:** Christiane M. Nyhsen, Laura J. Steinberg, Janice E. O’Connell

**Affiliations:** 1 Department of Radiology, Sunderland Royal Hospital, Sugarland Royal Hospital, Kayll Road, Sunderland, SR4 7TP UK; 2 Sunderland Royal Hospital, Sunderland, UK

**Keywords:** Radiology, Teaching, Student opinion, Teaching methods, Undergraduate medical education

## Abstract

**Objectives:**

To obtain medical students’ evaluation of the quality of undergraduate radiology teaching received, preferred teaching methods and resources. This is a follow-up project to an earlier study of junior doctors who felt that radiology teaching left them ill prepared for medical practice.

**Methods:**

A questionnaire to third and fifth year medical students undertaking clinical rotations at Newcastle University, UK.

**Results:**

The questionnaire was completed by 57/60 (95 %) of third and 37/40 (93 %) of final year medical students. Students received minimal radiology teaching in pre-clinical years, feeling this was insufficient. The majority of students rated interactive case-based teaching as effective. Self-directed learning resources such as textbooks, journals and even online learning modules were perceived as less effective. Other types of web resources rated higher. Motivation for most students when studying radiology was to achieve learning objectives needed to pass their next exams and/or to improve as a doctor.

**Conclusions:**

Medical students criticise the lack of radiology teaching in pre-clinical undergraduate years. Radiology teaching should be represented in all undergraduate years, preferably delivered via interactive teaching sessions. Currently available e-learning modules do not meet the students’ learning needs and there is a call for reliable, up-to-date open access electronic resources.

***Main Messages*:**

*• Radiology teaching should be represented in all pre-clinical and clinical undergraduate years.*

*• Medical students rate interactive case-based teaching sessions as very effective.*

*• There is a call for reliable, up-to-date open access electronic resources for medical students.*

## Introduction

The importance of undergraduate radiology teaching has been highlighted by recent surveys and publications from the European Society of Radiology, in particular the *White paper* [1–3]. In order to improve our radiology teaching delivery, we conducted a survey amongst medical students in our teaching hospital to learn their views on current teaching and preferred teaching methods and to gather comments on possibilities for improvement from the student perspective.

The undergraduate medical school curriculum at the Newcastle University, UK, lasts for 5 years. In the first 2 years teaching is “system based”, for example teaching of the cardiovascular system includes relevant anatomy, physiology, etc. There is no specific teaching on radiological anatomy. In the third year some basic radiology interpretation skills are taught, mainly concentrating on chest and abdominal radiographs. In the fourth year small group “problem-based learning” is undertaken for one term, including specialist imaging modalities relevant to the conditions under discussion. Thereafter students can select 36-week study modules, including a varying amount of radiology depending on the chosen specialty. The fifth year starts with specialty modules (like Gynaecology) containing very little radiology, followed by rotations in medicine and surgery where more advanced formal radiology teaching takes place as well as informal ward based teaching.

This article is a follow-up study to a survey undertaken amongst junior doctors, who felt that their earlier radiology teaching did not prepare them adequately for medical practice [4]. Results have already been published as an e-poster at the European Congress of Radiology in Vienna 2012 [5].

## Materials and method

During December 2010, paper questionnaires were distributed to all third and fifth year medical students from Newcastle University, UK, who were undertaking clinical rotations at Sunderland Royal Hospital.

The questionnaire included quantitative questions about the amount of radiology teaching received in their training to date and their perception of the quality of this teaching. It also covered a range of open questions regarding their preferred teaching methods and resources.

The students were asked to score each question using a Likert scale between 1 and 5, where 1 was ranked as poor or not effective and 5 was ranked as excellent or very effective. Regarding the use of teaching resources, “rarely used” was defined as less than once a term, “sometimes used” as once a month and “regularly used” as more than once a week.

There were also some free text questions allowing students to include further comments and suggestions.

## Results

### Response rate

Fifty-seven of 60 third year medical students (93 %) and 37 of 40 fifth year medical students (95 %) completed the questionnaire.

### Demographics

Ninety-one percent of third year students were aged between 19 and 24 years and 92 % of fifth year students were aged between 22 and 27 years. The relatively wide age distribution is due to the inclusion of mature students in the study cohort.

Fifty-three percent of the third year students were female (5 students did not complete this question) and 46 % of the fifth year students were female (100 % response rate).

### Frequency of teaching

All students highlighted the fact that radiology teaching was non-existent or very scarce in the first 2 years at medical school and over half of all students felt this was too little.

Students generally reported that they received teaching on a monthly or weekly basis in the third year. Interestingly, the majority (i.e. 70 %) of third years felt that this was adequate, whereas 68 % of fifth year students stated this was too little.

Teaching in the fourth year varies greatly because of individual student-selected components and elective periods; therefore assessment is difficult.

At the stage in the academic year when the questionnaire was completed, the fifth year students did not have formal radiology teaching. This takes place in the subsequent medical and surgical rotations, where they receive twice-monthly formal radiology teaching sessions. When questioned, fifth year students stated that they would like more sessions, in particular with experienced clinicians or radiologists.

### Type of teaching

**Fig. 1:**
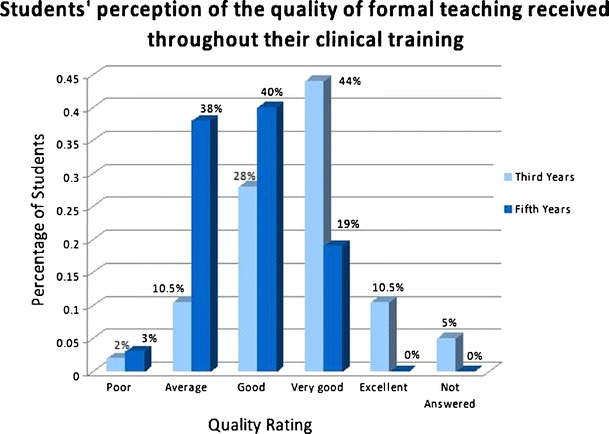
Students’ perception regarding formal teaching

Final year students reported having received more formal teaching than third year students, who stated more often an even mix between formal and informal teaching. Most students were happy with the quality of informal teaching received. The formal teaching received significantly different ratings from third and fifth year students, the older students being more critical about the quality of teaching (see Fig. 1).

**Fig. 2:**
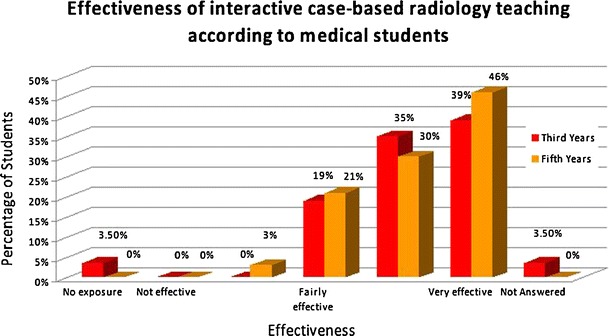
Effectiveness of interactive case-based teaching

Overwhelmingly, both the third and fifth year students felt that the most effective form of teaching was through *interactive case*-*based discussions* (see Fig. 2). The second most popular choice was interactive system-based teaching.

Other teaching methods attracted mixed ratings. “Presenting topics to a group” was not a popular choice, with the majority of students finding it “not at all” to only “fairly effective” (23 % of 3rd and 16 % of 5th year students had no exposure). “Exam style questioning” attracted almost a normal distribution of results with answers of or around “fairly effective”.

**Fig. 3:**
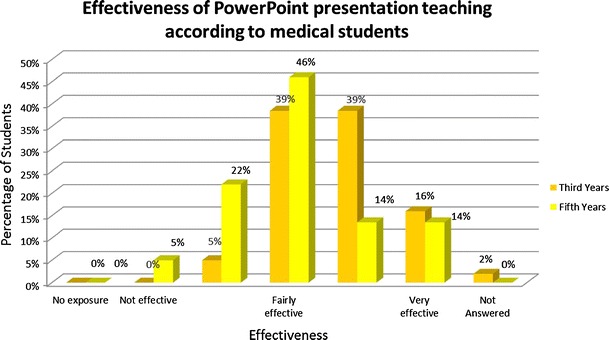
Effectiveness of PowerPoint presentation teaching

*PowerPoint presentations* were valued a little better with most students finding it “fairly” to “very effective”, third year students being more positive about this method than fifth year students (see Fig. 3). Radiology textbooks and journals were rarely used by medical students.

**Fig. 4:**
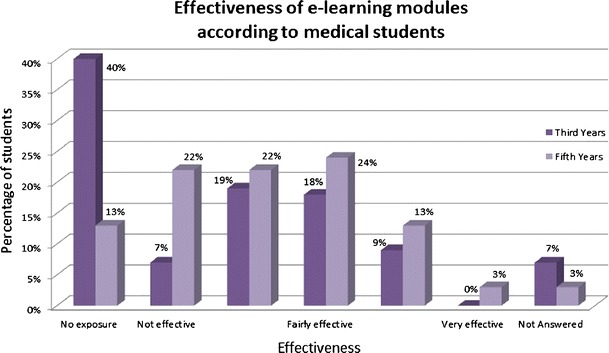
Effectiveness of e-learning modules

**Fig. 5:**
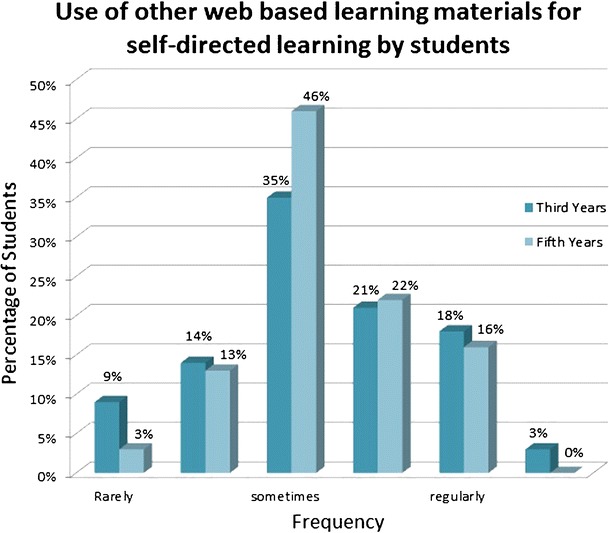
Use of other web-based learning materials

Interestingly, e-learning modules were rated relatively poorly and less effective than self-directed study from textbooks, whereby it has to be mentioned that third year students in particular had very little exposure to e-learning modules (see Fig. 4). Other web-based materials for self-directed learning were much more commonly accessed by both third and fifth year students, with little difference seen between the two groups (see Fig. 5). These included Google and Wikipedia as most commonly mentioned sources.

### Discrepancies between radiologists and other clinical teachers

Only a very small percentage of all students felt that there were discrepancies between the radiology teaching delivered by radiologists versus other clinicians (this is 7 % of 3rd year students and none of the 5th year students), with the differences noted in focus rather than content of taught sessions.

Free text comments described differences such as “clinicians taught at a more basic level” and “radiologist’s teaching aimed too high”, “radiologist’s teaching was broader on viewing” and “more detailed anatomy”. These comments are mainly from third year students.

### Motivation and radiology as career option

The vast majority of students stated that their primary motivation for studying radiology was passing the next exams and/or improving as a doctor. Seven percent of third year and 5 % of final year students study radiology because they are considering a career in the speciality.

A total of 3.5 % of third year students and 3 % of final year students feel that the teaching they have received has inspired them to consider a career in radiology.

### Teaching contents

Radiology teaching for the majority of fifth year students (95 %) included discussions regarding advantages/disadvantages of different imaging modalities such as ultrasound, CT and MRI and their indications/contraindications. Only 46 % of third years had received teaching on these topics.

### Thoughts for improvement

The free text comments commonly mention that shorter and more frequent sessions would be much better. Many students find that learning on cases with real patients aids information retention and makes teaching more interesting. Some students would value short assessments or a quiz to monitor their progress. Other students would welcome teaching on more complex imaging modalities including CT and MRI.

## Discussion

This study clearly demonstrates medical students’ interest in radiology but lack of early exposure to diagnostic imaging in their pre-clinical undergraduate years. The various benefits of introducing radiology early in the teaching curriculum have been demonstrated by several authors: Branstetter et al. showed in their initial study that students gain a higher opinion of radiology when this is taught in pre-clinical years [6] and that these attitudes also persist when graduating [7]. They furthermore highlight that better informed medical students are more likely to request appropriate diagnostic tests when they become clinicians and that this not only improves patient care but also the relationship between radiologists and future clinicians. In addition students may be more likely to choose radiology as an elective period, research topic or ultimately as a career [7, 8]. The latter may help in avoiding workforce shortages in areas such as breast imaging, as described by Roubidoux et al. [9].

Several studies describe the successful integration of radiology in the gross anatomical teaching curriculum [10–12]. Marker et al. reported that the digital radiology-based anatomy lectures were well received by students, aiding initial learning as well as anatomy exam preparation from home and showing a “trend toward some increase of interest in the field of radiology” [11]. Dettmer et al. described a new concept of teaching surgery, radiology and anatomy together, demonstrating the “direct relevance and applications for their future clinical work” to the students very early on in their studies and thus improving information retention and motivation [12]. The authors mention that students valued interdisciplinary learning as it added a new perspective on previously available anatomy teaching.

Another field where radiology can be successfully integrated in the pre-clinical curriculum is physiology, as demonstrated by Nay et al. [13]. The authors detail several examples of mainly radio-isotope case studies being used to teach normal physiology as well as pathophysiology.

In terms of teaching methods, medical students clearly preferred interactive case-based discussions over all other teaching methods such as presenting topics to a group or exam-style questioning. Interactive system-based discussions were the second most popular choice, which demonstrates that interactive elements in particular are favoured by students. Our previous study showed almost identical results in questioned junior doctors, confirming this trend amongst young learners [4].

Other authors have described very similar results. Zou et al. found that the majority of students preferred teaching with interactive dialogues, preferably in small groups with students volunteering to answer questions [14]. Students did however mention that some basic knowledge is needed to enable effective discussions and that some of these basic facts may be more efficiently covered in lectures.

Interactivity has been reported to subjectively improve concentration and enjoyment by Malek et al., with significantly better learning outcomes using case based teaching in radiology [15]. Ochoa and Wludyka suggested that interactive elements of their web-based teaching programme enhance students’ motivation as well as stimulate their ability in critical thinking [16]. Experienced teachers may simply argue that it is easier to keep students awake by maintaining the dialogue rather than finishing a PowerPoint monologue.

Our study shows that students do not perceive PowerPoint presentations negatively per se and in particular younger students actually value them. This shows that PowerPoint presentations are an effective teaching tool when used correctly. Interactive elements can be integrated easily into a slide show, as confirmed by Zou et al. [14]. Furthermore, there are audience response software packages available such as “Turning Point”, allowing active participation of students. We have used them in our education centre successfully. Nayak and Erinjeri highlight that such a system can supply the students with instant anonymous and non-threatening feedback, as well as giving students the opportunity to compare their answers with others in the group thereby increasing their confidence [17].

The medical students we questioned were not impressed by freely available e-learning modules and over a third of third year students had no exposure to online modules. This shows that either students have difficulties in finding appropriate web-based training resources themselves or the existing e-modules do not match the training needs of students. Many radiology resources are aimed at radiologists or postgraduate trainees. Furthermore, many of the existing teaching websites are somewhat dated, do not contain interactive elements and the quality of x-rays is not always comparable to digital PACS images that students are used to seeing on the wards. It is of some concern that students are relying on generic search engines such as Google and open access websites like Wikipedia to identify learning resources. This suggests that guidance should be offered to students to help them find suitable teaching websites. Alternatively dedicated e-learning modules have to be developed at the university hospitals where students receive training, an undertaking that we have not yet successfully completed ourselves.

Several authors have shown that e-learning modules can be successfully integrated into the undergraduate teaching curriculum. However, they have to be tailored to the specific training needs of medical students, aimed at the correct level, and they should ideally be as interactive as possible to maintain students’ interest and improve learning outcomes. Possibilities include covering basic facts with dedicated e-learning modules followed up with subsequent interactive case-based teaching sessions, ideally with a radiologist, where knowledge can be consolidated and any queries can be discussed. A similar model was used by Gotthardt et al. when their e-learning curriculum was introduced [18].

Students can furthermore be invited to participate in writing new e-learning material or updating current training files as demonstrated by Novak et al. [19]. This would ensure that the students’ perspective is at the core of the prepared teaching material. The motivation of students is an important consideration and asking students to participate in updating teaching files may increase their willingness to use it and revisit material. Students should ideally have home access to all teaching material, which has been mentioned as an important factor in several studies [11, 18]. Many teachers will argue that exams are one of the most effective motivating factors and that they would ensure a basic knowledge standard. Kourdioukova et al. strongly suggest these to be separate radiology exams so that radiology content is taken seriously and not neglected as a small percentage of questions in other modular exams [20].

In conclusion, we have shown that both undergraduate and postgraduate trainees feel that existing radiology teaching does not fully meet their learning needs or prepare them for clinical practice. Medical schools should be encouraged to introduce students to the basics of diagnostic imaging in the early undergraduate years, integrating this with system-based and case-based teaching during the pre-clinical and clinical phases of the curriculum. There is currently a lack of reliable, up-to-date electronic resources that are widely accessible to medical students and junior doctors. Co-operation between academic institutions should be encouraged in the development of validated open access radiological training resources that will meet the learning requirements of both undergraduate students and postgraduate trainees.
